# Supplementary material for: Increased Pathogenicity of the Nematophagous Fungus Drechmeria coniospora Following Long-Term Laboratory Culture
Source: Front Fungal Biol. 2021 Dec 16;2:778882. doi: 10.3389/ffunb.2021.778882 (PMC10512298; doi:10.3389/ffunb.2021.778882)
Supplement: Supplementary file 1 [file Table_1.PDF]

Supplementary Table 1: PCR tests used to genotype the *D. coniospora* Swe strains.

Strains to differentiate	Description	Target	Primer id	Sequence
Swe1, Swe2	Deletion in gene g1885.t1 (ODA83372.1)	Chr. 2, g1885.t1	F1_g1885	GTGGTCGACGAGGAACGC
			F2_g1885	CGGCTCAGTACAAAGCTACCC
			R_g1885	ACGACGTAGAAGAAGTCGCC
Swe1, Swe2	SNP in gene g8020.t1 (ODA75964.1): A1230G	Chr. 3, g8020.t1	F_g8020	ACCACCTACCACCTCTCGC
			R_g8020	GGCGACGATCTTGACGGG
Swe1, Swe2	SNP in gene g3072.t1 (ODA80305.1): C490A	Chr. 1, g3072.t1	F_g3072	ACCAGACGTTACTCCCAAGC
			R_g3072	ACCCTAAAGTTGTCCCAGAAGC
Swe2, Swe3	SNP in the gene g932.t1 (ODA82425.1): C1338T	Chr. 2, g932.t1	F_g932	AGTGCCCCAACAGGATTACC
			R_g932	ATGCGGTAGCCCATGAGATG
Swe2, Swe3	One insertion in gene g3500.t1 (ODA80732.1): 209insC	Chr. 1, g3500.t1	F_g3500	CATCATGCAAGGACACGCC
			R_g3500	CTGGGAGAGATGGGGGAGG
Swe2, Swe3	SNP in the gene g7471.t1 (ODA76724.1): G1486A	Chr. 3, g7471.t1	F_g7471	AAAAGACGGACTTTGGACGC
			R_g7471	GATTTTGTCGCTCGACTGGC

Target	Primers	Strain	Amplicon (bp)	Read out
g1885.t1	F1_g1885 + R_g1885	Swe1	403	Amplicon migration
	F1_g1885 + R_g1885	Swe2	383	
	F2_g1885 + R_g1885	Swe1	365	
	F2_g1885 + R_g1885	Swe2	0	
g8020.t1	F_g8020 + R_g8020	Swe1 / Swe2	282	Sequencing
g3072.t1	F_g3072 + R_g3072	Swe1 / Swe2	289	Sequencing
g932.t1	F_g932 + R_g932	Swe2 / Swe3	300	Restriction (*HhaI*) + migration
g3500.t1	F_g3500 + R_g3500	Swe2	249	Sequencing
	F_g3500 + R_g3500	Swe3	250	
g7471.t1	F_g7471 + R_g7471	Swe2 / Swe3	295	Sequencing

Supplementary table 2: Presence/absence of mitochondrial genes found in Swe2 compared to 3 closely related fungi mitogenomes

			Purpureocillium lilacinum	*Tolypocladium Inflatum*	*Tolypocladium cylindrosporum*	Swe2	
			10.1080/23802359.2019.1699466 (Li et al., 2020)	10.1007/s00253-017-8574-0 (Zhang et al., 2017)	10.1080/23802359.2020.1714495 (Zhang and Zhang 2020)	MITOS2 webserver, 10.1016/j.ympev.2012.08.023	MFannot webserver https://megasun.bch.umontreal.ca/cgi-bin/mfannot/mfannotInterface.pl
			MN635609.1	KY924883.1	MN842262.1	-	-
rRNA genes	small sub-unit	rns	ok	ok	ok	ok	ok
	large sub-unit	rnl	ok	ok	ok (2 introns; rps3 and GIY)	ok	ok
Protein-coding genes	ATP synthase	atp6	ok	ok	ok	ok	ok
		atp8	ok	ok	ok	ok	ok
		atp9	ok	ok	ok (1 intron; GIY)	ok	ok
	apocytochrome b	cob	ok	ok	ok (1 intron; GIY)	ok (1 intron; GIY)	ok (1 intron; GIY)
	cytochrome oxidase	cox1	ok	ok	ok (2 introns; LAGLIDADG and GIY))	ok (2 introns; LAGLIDADG and GIY)	ok (2 introns; LAGLIDADG and GIY)
		cox2	ok	ok	ok	ok	ok
		cox3	ok	ok	ok	ok	ok
	NADH dehydrogenase	nad1	ok	ok	ok	ok	ok
		nad2	ok	ok	ok	ok	ok
		nad3	ok	ok	ok	ok	ok
		nad4	ok	ok	ok	ok	ok
		nad4L	ok	ok	ok	ok	ok
		nad5	ok	ok	ok	ok (1 intron;LAGLIDADG)	ok (1 intron; LAGLIDADG)
		nad6	ok	ok	ok	ok	ok
	rps-like protein	rps3	ok	ok	ok	ok	ok
tRNA genes	trnA(tgc)		ok	ok	ok	ok	ok
	trnC(gca)		absent	ok	ok	ok	ok
	trnD(gtc)		ok	ok	ok	ok	ok
	trnE(ttc)		ok	ok	ok	ok	ok
	trnF(gaa)		ok	ok	ok	ok	ok
	trnG(acc)		absent	absent	ok	absent	absent
	trnG(tcc)		ok	ok	ok	ok	ok
	trnH(gtg)		ok	ok	ok	ok	ok
	trnI(gat)		ok	ok	ok	ok	ok
	trnK(ttt)		ok	ok	ok	ok	ok
	trnL(tag)		ok	ok	ok	ok	ok
	trnL(taa)		ok	ok	ok	ok	ok
	trnM(cat)		3 copies	3 copies	3 copies	3 copies	3 copies
	trnN(gtt)		ok	ok	ok	ok	ok
	trnP(tgg)		ok	ok	ok	ok	ok
	trnQ(ttg)		ok	ok	ok	ok	ok
	trnR(acg)		ok	ok	ok	ok	ok
	trnR(tct)		ok	ok	ok	ok	ok
	trnS(tga)		ok	ok	ok	ok	ok
	trnS(gct)		ok	ok	ok	ok	ok
	trnT(tgt)		absent	ok	ok	ok	ok
	trnV(tac)		absent	ok	ok	ok	ok
	trnW(tca)		ok	ok	ok	ok	ok
	trnY(gta)		ok	ok	ok	ok	ok

Supplementary table 3: Status in the new assembly of Swe2 scaffolds that had not previously been incoprorated into the genome assembly

Swe2 scaffold	length (nt)	presence of gene?	Insert in Swe2	Length after trimming	remark
JYHR01000012.1	173 380	yes	chromosome 3	160 167	
JYHR01000013.1	54 712	yes	chromosome 1	50 350	
JYHR01000014.1	48 392	yes	chromosome 3	43 976	
JYHR01000015.1	29 422	yes	chromosome 1	28 782	
JYHR01000016.1	28 571	yes	chromosome 3	28 571	
JYHR01000017.1	28 080	yes	chromosome 3	21 992	
JYHR01000018.1	27 206	yes	chromosome 3	27 206	
JYHR01000019.1	26 919	yes	chromosome 3	26 919	
JYHR01000020.1	26 539	yes	chromosome 2	26 539	
JYHR01000021.1	23 828	yes	mitochondria	NA	
JYHR01000022.1	23 058	no	NA	NA	
JYHR01000023.1	22 641	yes	chromosome 3	22 641	
JYHR01000024.1	20 036	yes	chromosome 3	16 817	
JYHR01000025.1	18 657	yes	chromosome 3	18 657	
JYHR01000026.1	16 902	yes	chromosome 3	16 902	
JYHR01000027.1	16 052	yes	chromosome 3	16 052	
JYHR01000028.1	14 597	no	NA	NA	
JYHR01000029.1	14 514	yes	chromosome 3	14 514	
JYHR01000030.1	14 170	yes	chromosome 2	14 170	
JYHR01000031.1	14 066	yes	chromosome 3	14 066	
JYHR01000032.1	9 307	yes	chromosome 3	9 307	
JYHR01000033.1	9 128	no	NA	NA	
JYHR01000034.1	8 345	no	NA	NA	
JYHR01000035.1	8 253	yes	chromosome 3	8 253	
JYHR01000036.1	6 725	no	NA	NA	
JYHR01000037.1	6 689	no	NA	NA	
JYHR01000038.1	6 421	yes	chromosome 3	6 421	
JYHR01000039.1	6 407	yes	NA	NA	Identical to JYHR01000038.1
JYHR01000040.1	5 655	no	NA	NA	
JYHR01000041.1	5 653	no	NA	NA	
JYHR01000042.1	5 624	no	NA	NA	
JYHR01000043.1	5 249	yes	NA	NA	Already present in the genome, assembly duplication
JYHR01000044.1	5 211	yes	chromosome 3	5 211	
JYHR01000045.1	4 839	no	NA	NA	
JYHR01000046.1	3 936	no	NA	NA	
JYHR01000047.1	3 817	yes	NA	NA	Already present in the genome, assembly duplication
JYHR01000048.1	3 440	no	NA	NA	
JYHR01000049.1	2 917	no	NA	NA	
JYHR01000050.1	2 571	no	NA	NA	
JYHR01000051.1	2 487	yes	chromosome 3	2 487	
JYHR01000052.1	2 111	no	NA	NA	
JYHR01000053.1	2 040	no	NA	NA	
JYHR01000054.1	1 554	no	NA	NA	
JYHR01000055.1	1 460	no	NA	NA	
JYHR01000056.1	1 434	no	NA	NA	
JYHR01000057.1	1 343	no	NA	NA	
JYHR01000058.1	1 318	no	NA	NA	
JYHR01000059.1	1 285	no	NA	NA	
JYHR01000060.1	1 146	no	NA	NA	
JYHR01000061.1	1 041	no	NA	NA	
JYHR01000062.1	932	no	NA	NA	
JYHR01000063.1	902	no	NA	NA	
JYHR01000064.1	891	no	NA	NA	
JYHR01000065.1	825	no	NA	NA	
JYHR01000066.1	814	no	NA	NA	
JYHR01000067.1	812	no	NA	NA	
JYHR01000068.1	757	no	NA	NA	
JYHR01000069.1	722	no	NA	NA	
JYHR01000070.1	701	no	NA	NA	
JYHR01000071.1	636	no	NA	NA	
JYHR01000072.1	584	no	NA	NA	
JYHR01000073.1	556	no	NA	NA	
JYHR01000074.1	536	no	NA	NA	
JYHR01000075.1	505	no	NA	NA	

Supplementary table 3 (continued): Swe2 assembly statistics

	Chromosomes	Unplaced scaffolds
Genome size (Mb)	31.75	0.128
Sequences	3 + MT	38
GC content (%)	55.25	44.38
% of unknowns (Ns)	0.11	3.8
No. predicted protein-coding genes	8702	0

Supplementary table 4: ANI values for all pairs of Swe genomes from pyANI v0.29. Sequences were aligned with MUMmer (ANIm) or BLAST+ (ANIb). Tetranuccleotides correlations represent similarity with alignement-free method.

Measure	Query strain	Swe1	Swe2	Swe3
ANIm	Swe1	100	99.9052	99.9896
	Swe2	99.9052	100	99.9060
	Swe3	99.9896	99.9060	100
ANIb	Swe1	100	99.7694	99.9901
	Swe2	99.9155	100	99.9154
	Swe3	99.9820	99.7608	100
Tetranuccleotides correlations	Swe1	100	99.9709	99.9971
	Swe2	99.9709	100	99.9550
	Swe3	99.9971	99.9550	100

Supplementary Table 5: Results of BLAST searches of Dan2 sexual reproduction-related proteins

Query	Query ID	Query size (aa)	Best BLASTP hit in Swe2	Score	%ID	Alignment length (aa)	Query coverage (%)	Comment
MAT1-1-1	KYK59754.1	370	-	-	-	-	-	
MAT1-1-2	KYK59755.1	353	-	-	-	-	-	
MAT1-1-3	KYK59756.1	166	ODA81607.1	71	40	75	45	Alignment is restricted to the protein domain (HMG-box - PF00505)
RIP - rid1	KYK59907.1	646	ODA78704.1	776	91	416	64	
RIP - dim-2	KYK56049.1	377	ODA76538.1	598	86.4	397	100	
Late sexual development protein	KYK59150.1	355	ODA77900.1	706	99.72	355	100	
Heterokaryon incompatibility	KYK58811.1	804	ODA84177.1	1630	98.88	804	100	
Heterokaryon incompatibility	KYK56754.1	938	ODA78419.1	1898	99.68	938	100	
Heterokaryon incompatibility	KYK56328.1	752	ODA76785.1	1506	99.73	732	97	
Heterokaryon incompatibility - putative	KYK55209.1	749	ODA82169.1	1546	99.73	749	100	

TBLASTN with e-value threshold 0.00000000001. Dataset used for the TBLASTN searches: https://www.uniprot.org/uniprot/?query=gene%3Amat1-2-1+OR+gene%3Amat1-2-2+OR+gene%3Amat1-2-3+OR+gene%3Amat1-1-1+OR+gene%3Amat1-1-2+OR+gene%3Amat1-1-3+&sort=score

Protein of interest	Target	Best HSP (TBLASTN)	Best HSP localization	Best Query ID (UniProt ID)	Comment
MAT1-1-1	Swe2	no hit			
MAT1-1-2	Swe2	no hit			
MAT1-1-3	Swe2	ODA78559.1	Chr. 1	A7KPA5_COCPO	Querry does not have the required protein domain (HMG-box - PF00505)
MAT1-2-1	Swe2	not hit	Chr. 1	V9LW71_9HYPO	
MAT1-2-2	Swe2	ODA81607.1	Chr. 2	A0A2K8C394_9PEZI	Alignment is restricted to the protein domain (HMG-box - PF00505)
MAT1-2-3	Swe2	not hit			
MAT1-1-1	Dan2	KYK59754.1	Chr. 1 (CM004174.1)	U3N6X4_TOLIN	Presence of the protein domain "Mating-type protein MAT alpha 1 HMG-box" (PF04769)
MAT1-1-2	Dan2	KYK59755.1	Chr. 1 (CM004174.1)	Q1MVS6_TOLIN	Presence of the protein domain "Mating type protein 1-1-2" (PF17043)
MAT1-1-3	Dan2	KYK59757.1	Chr. 1 (CM004174.1)	A7KPA5_COCPO	Querry does not have the required protein domain (HMG-box - PF00505)
		KYK59756.1	Chr. 1 (CM004174.1)	Q1MVS7_TOLIN	Presence of of the protein domain "HMG-box" (PF00505)
MAT1-2-1	Dan2	KYK55799.1	Chr. 3 (CM004176.1)	K4H8Z2_9HELO	Alignment is restricted to the protein domain (HMG-box - PF00505)
MAT1-2-2	Dan2	KYK55799.1	Chr. 3 (CM004176.1)	A0A2K8C394_9PEZI	Alignment is restricted to the protein domain (HMG-box - PF00505)
MAT1-2-3	Dan2	no hit			

Supplementary table 7: RNAseq read count on the Swe2 target genes

The data from Dan2 were aligned on the Swe2 genome, with TopHat v2.1.1. The raw read counts were obtained with the command *samtools view -F 2304 <BAMfile> | wc -l*

	Swe2 data				Dan2 data			
	Lebrigand et al. 2016 Sup. tab. 13		Lebrigand et al. 2016 Sup. tab. 12		Zhang et al. 2016			
	5 h (p.i.)	12 h (p.i.)	Mycelia	Spores	Mycelia 1	Mycelia 2	Infection	Ratio Infection/Mycelia*
fg4743.t1	0	0	11	16	60	105	3	0.22
g1354.t1	0	0	1064	1429	206	478	157	2.80
g3072.t1	1	0	96	75	131	323	198	5.31
g8020.t1	0	0	447	558	593	1791	197	1.01
g1885.t1	0	0	1210	1123	723	1637	289	1.49
g7143.t1	0	0	334	391	809	1575	349	1.78
g7471.t1	0	0	502	700	283	646	204	2.68
g7915.t1	0	0	1102	1592	944	2162	102	0.40
g932.t1 (DcSre1)	1	0	751	1048	1638	4478	892	1.78
g3500.t1 (DcBud2)	1	0	274	323	369	948	1574	14.56
Num. of read	n.a.	n.a.	48 624 490	48 695 314	22 917 017	52 669 623	12 408 106	n.a.

* Ratio normalised by the number of read present in the 3 datasets
