# Supplementary material for: Increased Pathogenicity of the Nematophagous Fungus Drechmeria coniospora Following Long-Term Laboratory Culture
Source: Front Fungal Biol. 2021 Dec 16;2:778882. doi: 10.3389/ffunb.2021.778882 (PMC10512298; doi:10.3389/ffunb.2021.778882)
Supplement: Supplementary file 2 [file Image_1.PDF]

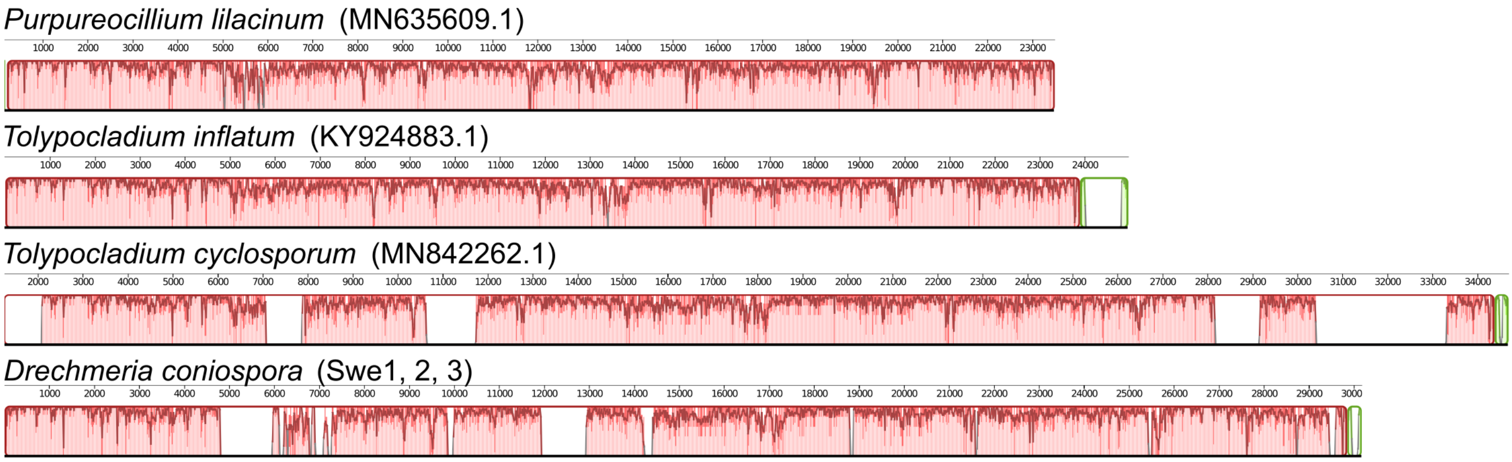

Supplementary Figure S1: Alignment of mitochondrial genomes from *D. coniospora* and 3 closely related fungi. For each species, a histogram of the overall sequence conservation (from 0 to 100%) calculated using a sliding window of fixed size is shown under the genome coordinates (in kb). The regions in red are syntenic and conserved across the four species, those in green correspond to sequences absent from *P. lilacinum*.

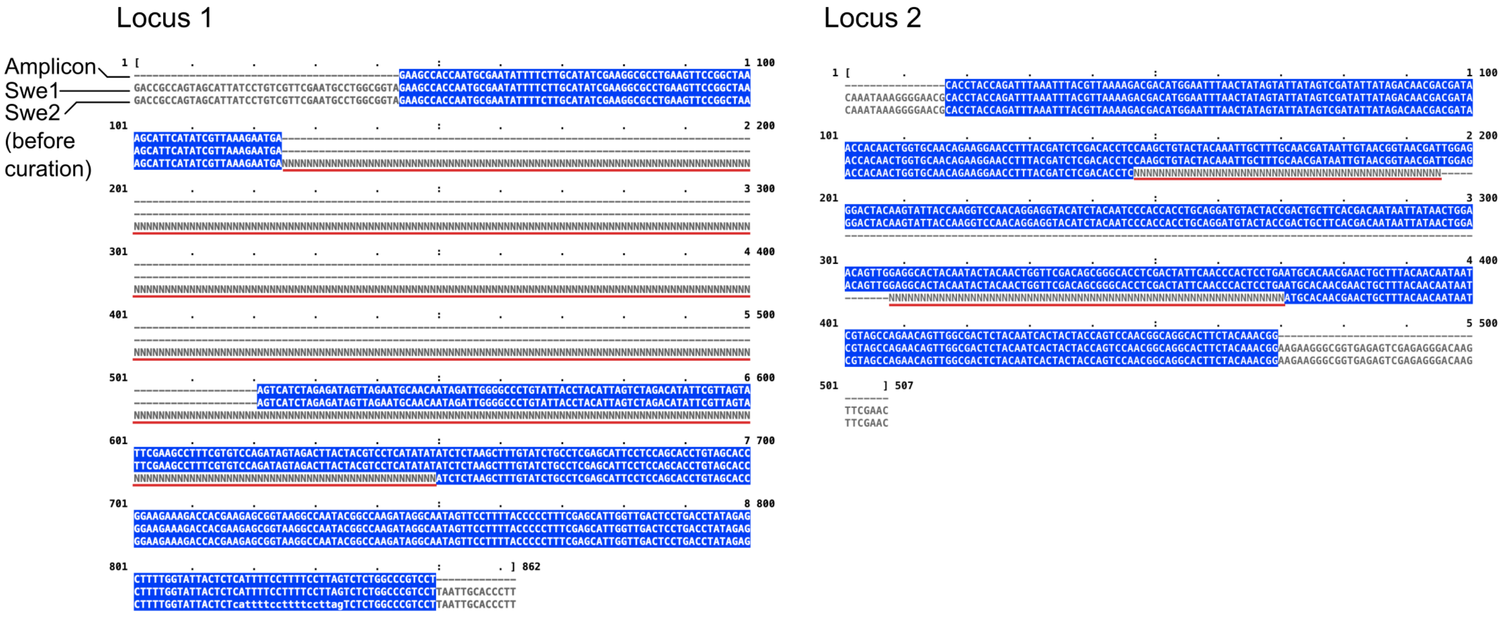

Supplementary Figure S2: Experimental validation of strategy to replace undetermined Swe2 sequences. Sequence alignments of 2 sequenced amplicons from Swe2, and the corresponding regions from the Swe1 and existing Swe2 genomic sequences. The blue highlights matching positions between the 3 sequences. The red lines indicate the stretches of underdetermined bases in the original Swe2 genome. Note, these regions were chosen as the corresponding sequence in Swe3 is identical to that in Swe1. The primers pairs used were GACCGCCAGTAGCATTATCC and AAGGGTGCAATTAAGGACGG for the left locus; CAAATAAAGGGGAACGCACC and GTTCGAACTTGTCCCTCTCG for the right.

Swe2 chromosome 3: 6974792..6974831

g4221.t1

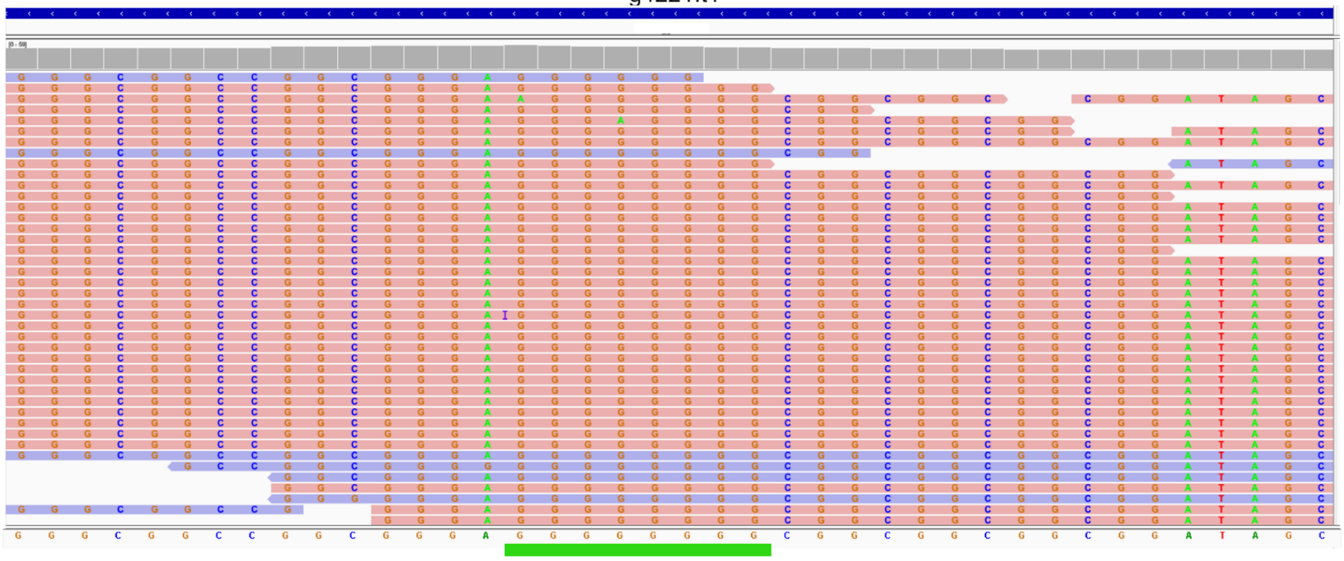

Swe3 chromosome 3: 7109562..7109603

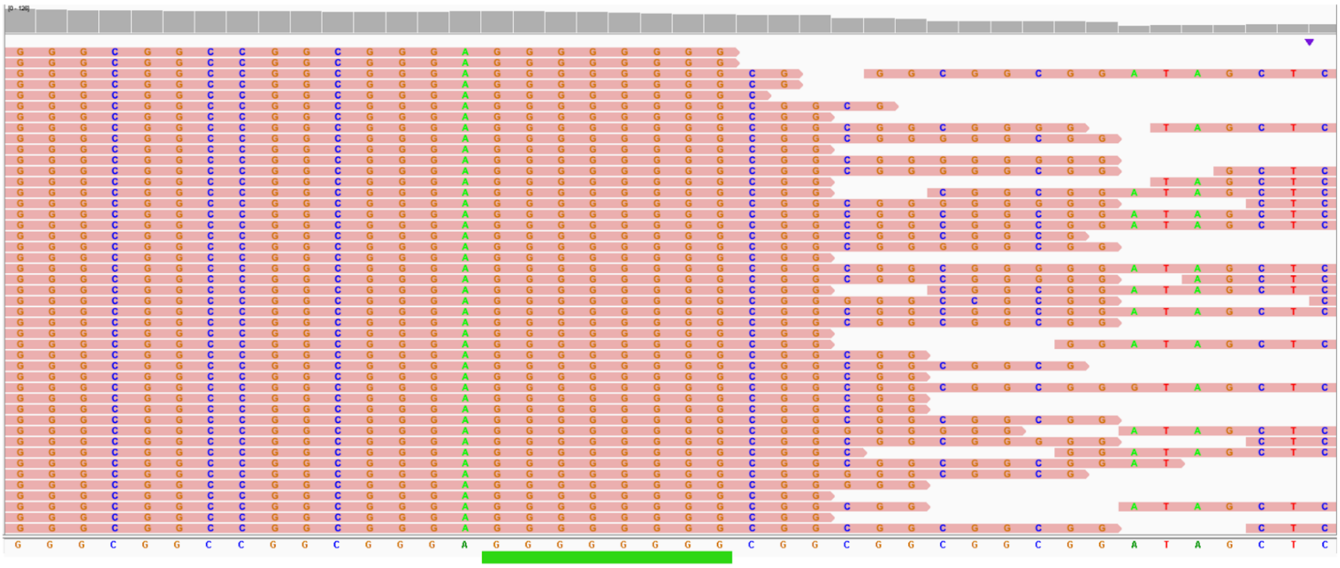

Swe1 chromosome 3: 7099070..7099100

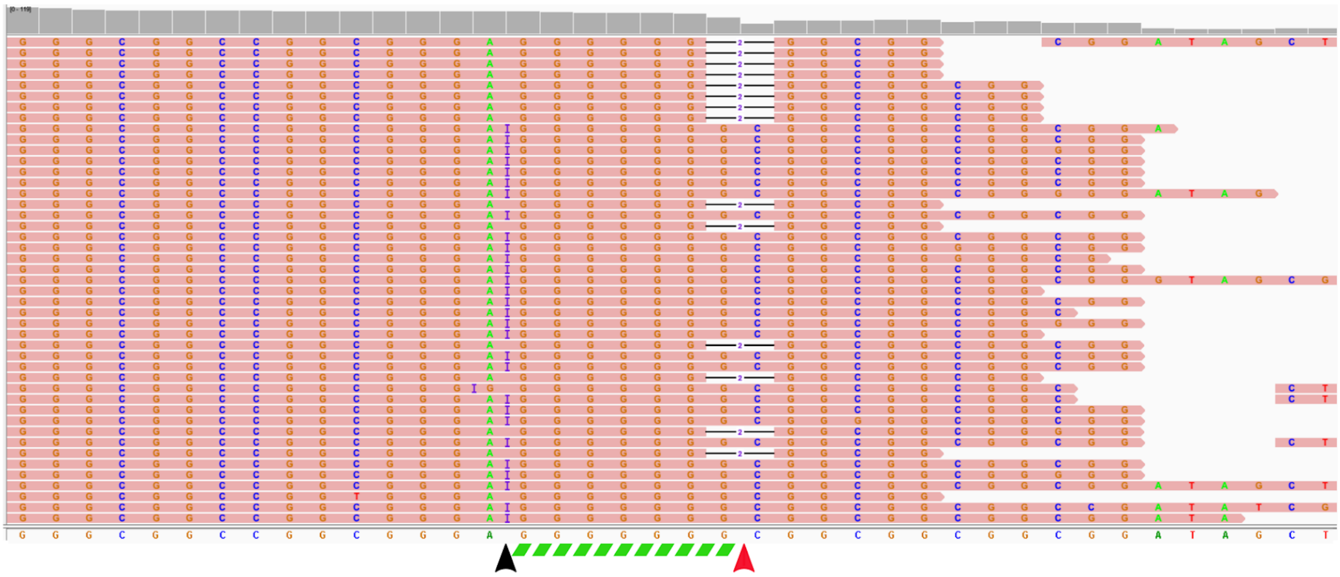

Supplementary Figure S3: Homopolymeric region bearing a residual sequence error.
Mapping of short DNA sequencing reads to the equivalent locus in the Swe2, Swe3 and Swe1 genomes, for which the respective genomic coordinates are indicated. A selection of the reads for each is represented, with their sequences in a salmon or light blue background, depending on their orientation. The sequence predicted for the locus, after polishing for Swe1 and Swe3 (Courtine et al. 2020), is shown below the mapped reads in each panel. The green rectangles under the top and middle panels highlight a homopolymer of 8 Gs. This 8 G sequence, supported by a large majority of short reads in all cases, has been incorrectly truncated to 7 Gs in Swe1, as highlighted by the dashed green rectangle. The black arrowhead marks the position where numerous reads have an extra G (an insertion as indicated by the blue I) that has not been taken into proper consideration, and the red arrowhead the highlights a position where numerous reads have been mismapped because of the tandem repetition of the sequence CGG, 3’ to the homopolymeric sequence, resulting in the inference of 2 nucleotide deletions, with respect to the (erroneous) reference, as indicated by -2-.

A

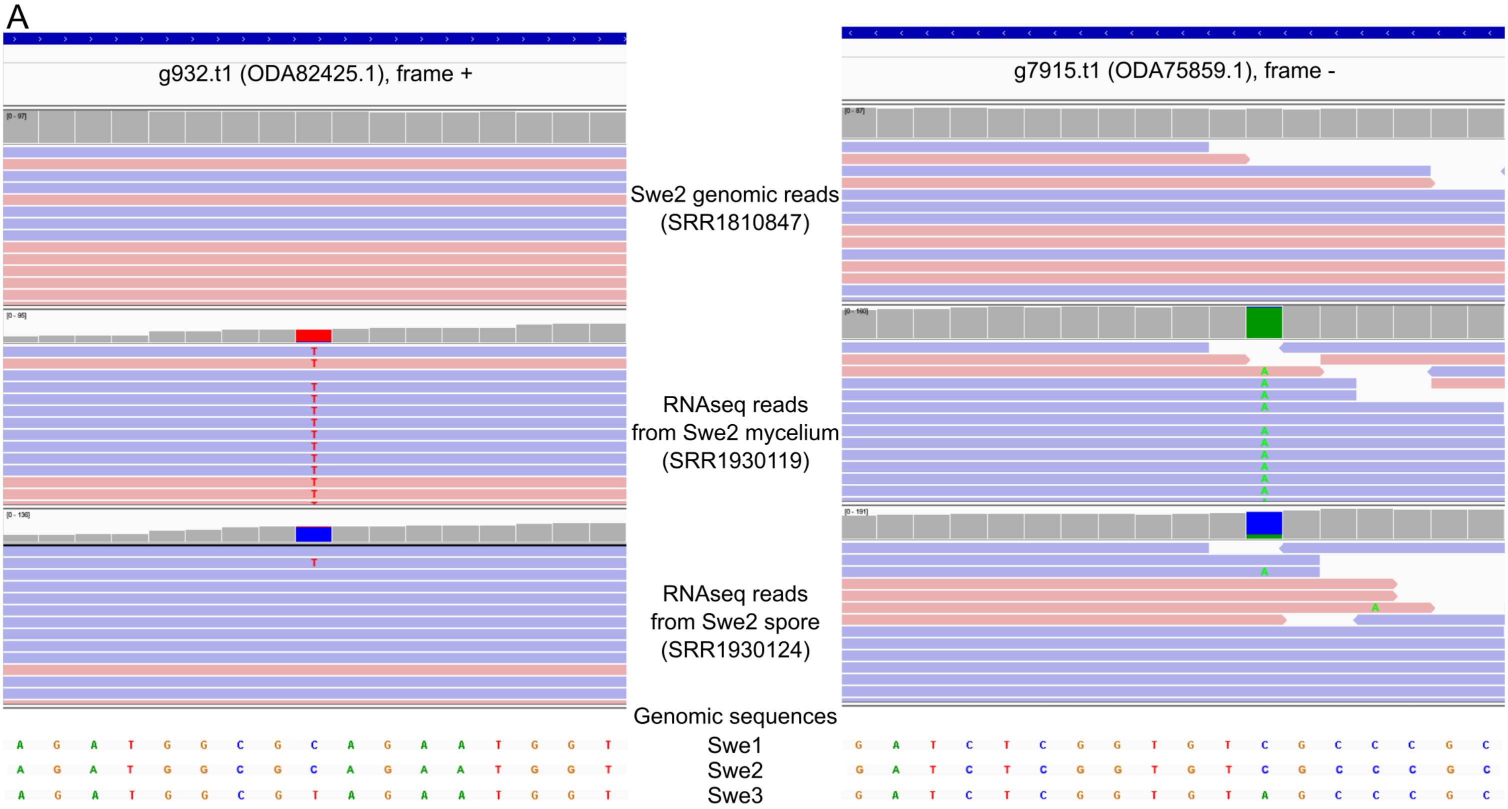

B

Gene	Allele	Genomic reads	Mycelium RNAseq	Spores RNAseq
g932.t1	Ref (C)	91 (99%)	3 (8%)	59 (92%)
	Alt (T)	1 (1%)	34 (92%)	5 (8%)
g7915.t1	Ref (C)	74 (100%)	5 (3%)	128 (82%)
	Alt (A)	0	144 (96%)	28 (18%)

Supplementary Figure S4: Examples of sequence heterogeneity captured by RNAseq. (**A**) Evidence at two sites for a disparity between the Swe2 genomic sequence and the sequence determined by transcriptome sequencing. Screen captures from IGV showing the mapping of genomic reads (top), and two RNAseq read sets from mycelia (middle) and spores (bottom). The boxes at the top of each panel represent the read coverage at each nucleotide position. They are coloured grey if >95% of reads support the consensus sequence for the Swe2 genome (shown below; identical to the corresponding sequence in the Swe1 genome). If an alternative nucleotide is supported by at least 5% of reads, the boxes are coloured proportionately, with the colours corresponding to those used for the different nucleotides in the genomic sequence shown below. Reads are represented in salmon or light blue, depending on their orientation. In those that do not match entirely the genomic sequence, non-consensus nucleotides are shown. For g7915.t1 (right), the gene is in the 3'-5' orientation, while the alleles are given for the 5'-3' sequence. (**B**) Summary of the number of reads supporting each allele in the 3 sets of reads.

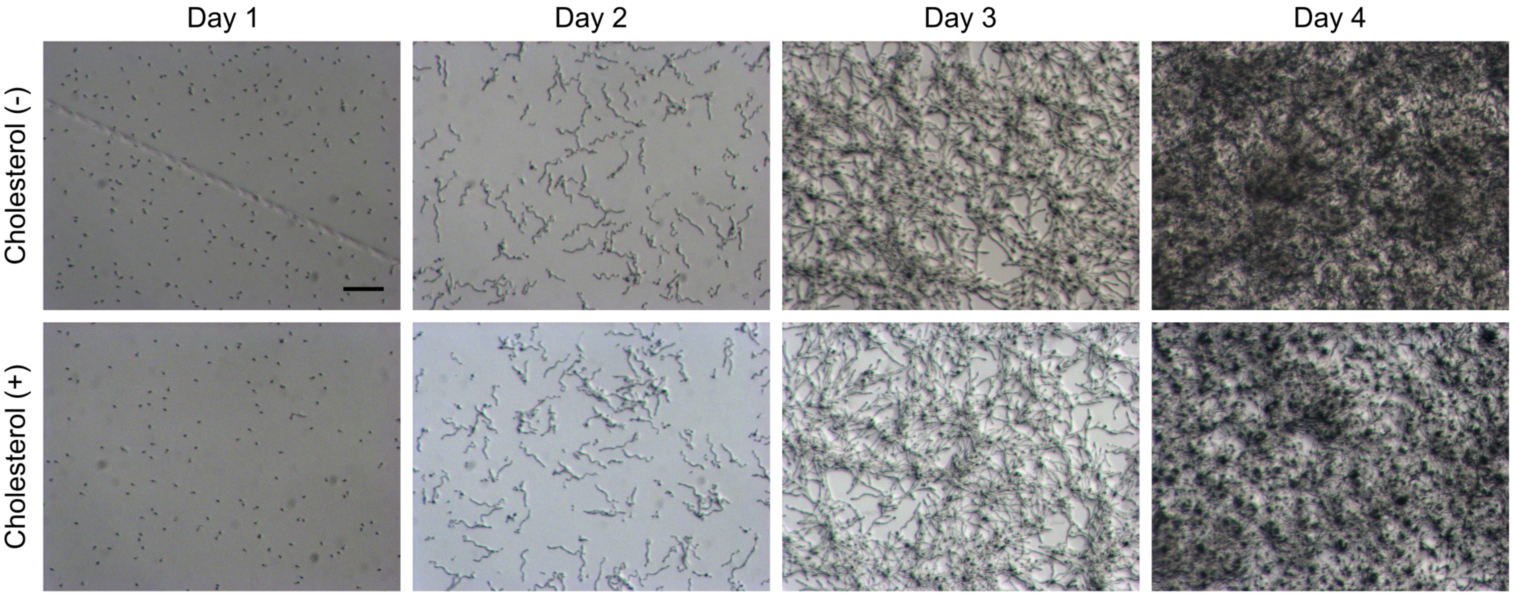

Supplementary Figure S5: Growth of a *DcSre1* mutant is not altered by cholesterol supplementation. Swe3 carries a null allele of *DcSre1*. Fungal growth on standard NGM plates (+) or plates without (-) the normal 5 µg/l cholesterol supplementation was monitored at the indicated times. Scale = 100 µm.

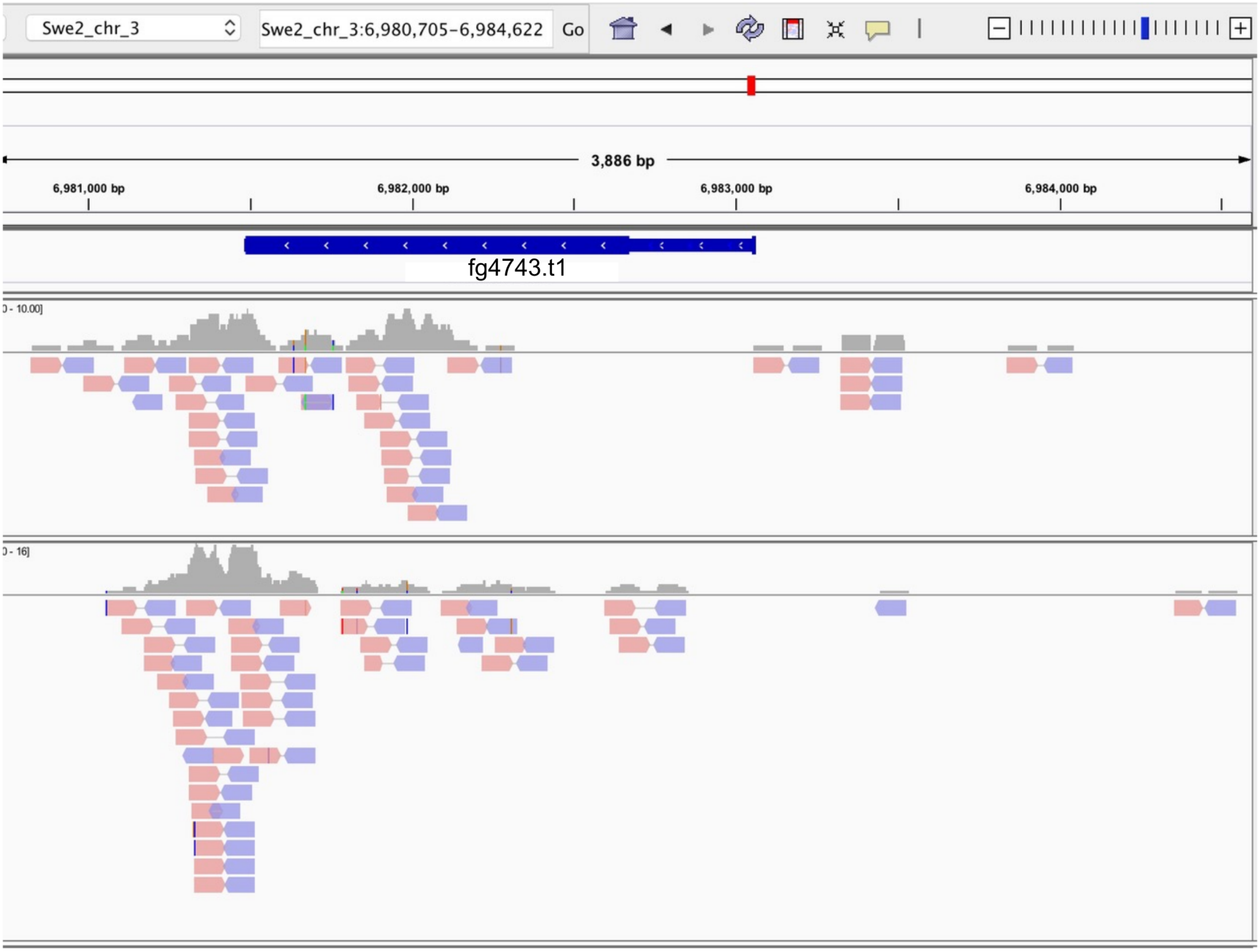

Supplementary Figure S6: Imperfect RNAseq support for the current gene model of fg4743. Screen capture from IGV showing the mapping of two RNAseq read sets from mycelia (top) and spores (bottom) at the fg4743 (ODA79924.1) locus. The boxes at the top of each panel represent the read coverage at each nucleotide position. They are coloured grey if >95% of reads support the consensus sequence for the Swe2 genome. If an alternative nucleotide is supported by at least 5% of reads, the boxes are coloured proportionately, with the colours being the standard ones for the different nucleotides. Reads are represented in salmon or light blue, depending on their orientation, with coloured vertical lines indicating non-consensus nucleotides; paired reads are connected by a thin horizontal line.
